# MT1-MMP Inhibits the Activity of Bst-2 via Their Cytoplasmic Domains Dependent Interaction

**DOI:** 10.3390/ijms17060818

**Published:** 2016-05-26

**Authors:** Long Fan, Li Liu, Cuicui Zhu, Qingyi Zhu, Shan Lu, Ping Liu

**Affiliations:** 1 Jiangsu Province Key Laboratory for Molecular and Medicine Biotechnology, College of Life Sciences, Nanjing Normal University, Nanjing 210023, China; fl891116@163.com (L.F.); zhu_ccyx_12@163.com (C.Z.); lu_shan@hotmail.com (S.L.); 2 Laboratory of Molecular Biology, Jiangsu Province Hospital of Traditional Chinese Medicine, Nanjing 210029, China; jsszyyfz@126.com; 3 Department of Urology, Jiangsu Province Hospital of Traditional Chinese Medicine, Nanjing 210029, China; zhuqy1971@126.com

**Keywords:** bone marrow stromal cell antigen 2 (Bst-2), Membrane type-1 matrix metalloproteinase (MT1-MMP), virus release, tetherin

## Abstract

Bst-2 (bone marrow stromal cell antigen 2) is a type II membrane protein, and it acts as a tetherin to inhibit virion releasing from infectious cells. Membrane type-1 matrix metalloproteinase (MT1-MMP) is a protease. It plays a pivotal role in cellular growth and migration by activating proMMP-2 into active MMP2. Our results here elaborate that MT1-MMP inhibits the tetherin activity of Bst-2 by interacting with Bst-2, and the cytoplasmic domains of both Bst-2 and MT1-MMP play critical roles within this interaction. Based on our experimental data, the assays for virion release and co-immunoprecipitation have clearly demonstrated that the activity of Bst-2 is markedly inhibited by MT1-MMP via their interaction; and both the N-terminal domain of Bst-2 and the C-terminal domain of MT1-MMP are important in the interaction. Immunostaining and Confocal Microscopy assay shows that MT1-MMP interacts with Bst-2 to form granular particles trafficking into cytoplasm from membrane and, finally, results in Bst-2 and MT1-MMP both being inhibited. In addition, mutant experiments elucidate that the N-terminal domain of Bst-2 is not only important in relating to the activity of Bst-2 itself, but is important for inhibiting the MT1-MMP/proMMP2/MMP2 pathway. These findings suggest that MT1-MMP is a novel inhibitor of Bst-2 in MT1-MMP expressed cell lines and also indicate that both the N-terminal domain of Bst-2 and the C-terminal domain of MT1-MMP are crucial in down-regulation.

## 1. Introduction

Bst-2 (bone marrow stromal cell antigen 2, also called tetherin, CD317, or HM1.24) is a type II transmembrane protein with a unique topology including two different types of membrane anchors, an N-terminal transmembrane anchor and a putative C-terminal glycophosphatidylinositol (GPI) lipid anchor [1,2]. It is found that Bst-2 is a host cell “restriction factor” counteracted by the HIV-1 accessory protein Vpu to limit the spread of enveloped viruses, and furthermore, to interfere with the release step of the replication cycle of viruses through tethering virion to infected cells, thus efficiently inhibiting the replication of HIV-1 [3,4]. Structurally, Bst-2 contains a short, 21-amino-acid cytoplasmic N-terminal tail (NT), followed by an α-helical transmembrane (TM) domain, a predominantly helical extracellular domain (EC) with an extended parallel coiled-coil, and a C-terminal GPI component. The cytoplasmic domain (N-terminal domain, NT domain) of Bst-2 plays key roles in its shuttling back and forth between cytoplasm and membrane as well as in its interacting with Vpu protein to tether virion onto cell surfaces [5,6]. In addition, Bst-2 can also interact/bind with other proteins via its NT domain to regulate the activities and bio-functions of other proteins [7,8].

Membrane-type matrix metalloproteinase 1 (MT1-MMP, also MMP14), a type I membrane protein belonging to the MT-MMP family, is one of the key proteins involved in many physiological and pathological processes, from normal cell development to cancer cell growth and metastasis [9,10,11,12]. The main function of MT1-MMP is identified as a cell surface proteinase to display multifunctions in degrading the ECM components, including type I and II collagen, fibronectin, vitronectin, laminin, fibrin and proteoglycan [13,14]. Usually, cells utilize MT1-MMP to change and modify their surrounding environment in many bio-processes such as angiogenesis, tissue remodeling, tumor invasion and metastasis [15,16,17]. The activity of MT1-MMP is regulated in three different ways: the first is that it is inhibited by its endogenous inhibitors, such as TIMP-2 and TIMP3 [18,19]; the second is via protein trafficking between cytoplasm and plasma membrane [20,21,22,23,24,25]; and the third is regulation in its expression levels, such as the transcription of MT1-MMP being regulated by the Wnt-β-catenin-Tcf4 signal pathway [26,27,28].

Previously, we have reported that Bst-2 inhibits MT1-MMP activity through their interaction and results in decreasing the activation of proMMP2 in MDCK (Madin-Darby canine kidney cells) and HT1080 (human fibrosarcoma cells) cell lines [29]. In this study, we still use these two cell lines as the study cell models. (It is because MDCK cells have no endogenous MT1-MMP and Bst-2 expression, while HT1080 cells have a low endogenous expression level of these two proteins). Our results demonstrate that MT1-MMP reversely inhibits the tetherin activity of Bst-2 also, through the interaction between themselves. The cytoplasmic tails of both MT1-MMP (C-terminal domain) and Bst-2 (N-terminal domain, NT-domain) are key domains in the interaction between these two proteins and also play critical roles in down-regulating the activity and bio-function of Bst-2. Furthermore, we find that the N-terminal domain of Bst-2 is important not only in regulating Bst-2 activity, but in regulating the activity of MT1-MMP. In addition, our data show that the transcription and protein levels of Bst-2 are not affected by MT1-MMP. Taken together, membrane protein MT1-MMP inhibits the activity and bio-function of membrane protein Bst-2 just through the dependent interaction of cytoplasmic tails (N-terminal domain of Bst-2 and C-terminal domain of MT1-MMP), and *vice versa*. This result may provide some positive clues for inhibiting enveloped virion infection and treating tumor metastasis in the clinic.

## 2. Results

### 2.1. Induced Expression of Bst-2 in HT1080 Cells and Its Activity Inhibition by MT1-MMP in Both MDCK and HT1080 Cells

**Figure 1:**
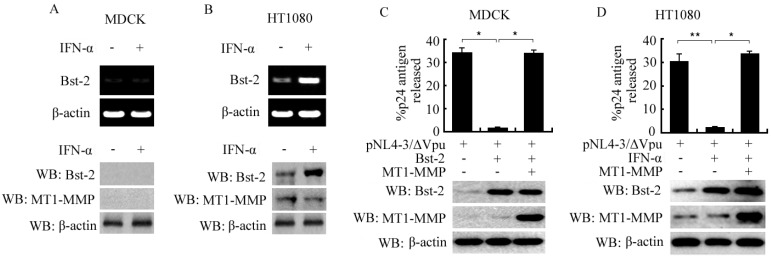
Expression of Bst-2 and its activity inhibition by MT1-MMP in MDCK cells and HT1080 cells. (**A**,**B**) Cells seeded in 6-plates were treated with or without IFN-α; and then mRNA and protein levels of Bst-2 were assayed using RT-PCR and western-blot; (**C**,**D**) cells were divided into two parts and seeded in 6-plates overnight, plasmids pNL4-3/ΔVpu, Bst-2 and MT1-MMP were transfected or co-transfected in HT1080 cells and MDCK cells as described in figures; 48 h after transfection, one part culture for Bst-2 activity assay, the fractional release of p24 capsid was determined as the concentration of p24 antigen in the supernatants divided by the total concentration of p24 antigen in both the supernatants and cell lysates; the other part of the cells was for western-blot assay. * *p* < 0.01; ** *p* < 0.05.

MDCK and HT1080 cells were seeded into 6-well plates and treated with or without IFN-α, as indicated in Figure 1A,B. Twenty-four hours after treatment, cells were harvested for RT-PCR and western-blot assay. The data showed that Bst-2 was not expressed in MDCK cells with or without treatment of IFN-α in both mRNA and protein levels; whereas in HT1080 cells, Bst-2 was a little originally expressed and much more inducibly expressed by IFN-α in both mRNA and protein levels (Figure 1A,B).

To test the effect of MT1-MMP on the tetherin activity of Bst-2, MDCK and HT1080 cells were seeded in 6-well plates and transfected or co-transfected with plasmid, as in Figure 1C,D. Forty-eight hours later, culture supernatants and cells were harvested and virion release was assayed as described in “Materials and Methods”. From our data, the fractional HIV-1 p24 capsid antigen release of cells with pNL4-3/ΔVpu was much more decreased by transfected Bst-2 in MDCK cells and by IFN-α-induced Bst-2 in HT1080 cells, and then rescued by co-transfected MT1-MMP in both MDCK and HT1080 cells (Figure 1C,D). In addition, we found that the effect of knocking down MT1-MMP with siMT1-MMP on the tetherin activity of Bst2 was not so obviously in HT1080 cells (because of the low expression of MT1-MMP. Shown as Figure S1).

All of results demonstrated that Bst-2 inhibited the release of virus from infectious cells and this inhibition could be reversed by MT1-MMP, which meant that Bst-2 tetherin activity was blocked by MT1-MMP.

### 2.2. Expression of Bst-2 Including mRNA Level and Protein Level Was Not Affected by MT1-MMP

**Figure 2:**
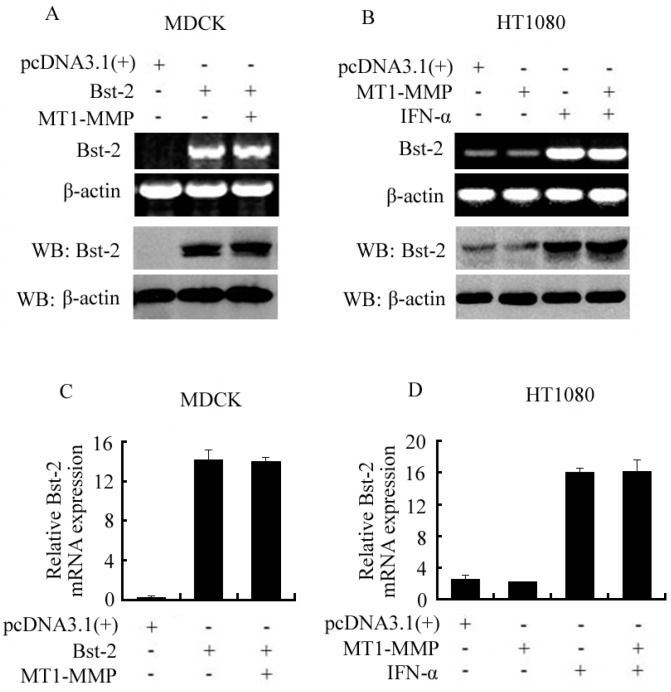
Effect of MT1-MMP on the expression of Bst-2 in mRNA and protein levels HT1080 and MDCK cells. Cells were divided into two parts and seeded in 6-plates overnight, then transfected with plasmids as shown in figures; 48 h after transfection, (**A**,**B**) one part of the cells was treated with TRIZOL for RT-PCR assay and lysed with lysis buffer for western-blot assay; (**C**,**D**) the other part of the cells was treated with TRIZOL and harvested for qPCR as described in “Materials and Methods”.

Cells seeded in 6-well plates were cultured overnight and then transfected with plasmids and treated with IFN-α as shown in Figure 2. After transfection for 48 h and treatment for 24 h with IFN-α, cells were collected for RT-PCR, western-blot assay and qPCR. From RT-PCR and Western-blot data, transient expression of Bst-2 was not affected by transiently over-expressed MT1-MMP in MDCK cells (Figure 2A), and also expression of endogenous Bst-2 (with or without treatment of IFN-α) was not changed by transiently over-expressed MT1-MMP in HT1080 cells (Figure 2B). All the expression of Bst-2 here included both mRNA and protein levels. Furthermore, our qPCR results also showed that the transient transcription (mRNA levels) of Bst-2 was not changed with the transiently over-expressed MT1-MMP in MDCK cells, and also the transcription of endogenous Bst-2 (with or without treatment of IFN-α) was not affected by transiently over-expressed MT1-MMP in HT1080 cells (Figure 2C,D).

These results demonstrated that MT1-MMP could not affect the expression of the *Bst-2* gene in both mRNA and protein levels, and indicated that MT1-MMP inhibiting Bst-2 activity was not via down-regulating the expression of the *Bst-2* gene.

### 2.3. Interaction and Co-Localization Happened between Proteins Bst-2 and MT1-MMP

**Figure 3:**
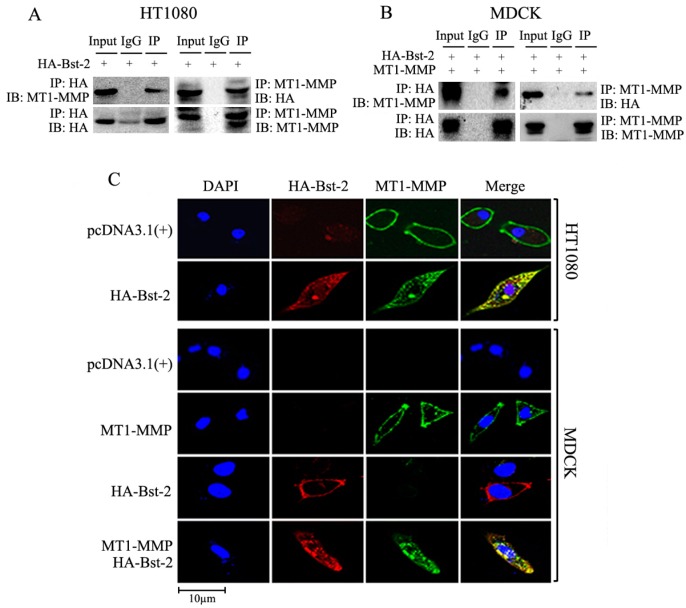
MT1-MMP interacted and co-localized with Bst-2 in HT1080 and MDCK cells. (**A**,**B**) Cells were cultured in 10-cm dishes and transfected with HA-Bst-2 (HT1080 cells) and co-transfected HA-Bst-2 with MT1-MMP (MDCK cells) as indicated in figures; 48 h after transfection, cells were harvested and lysed for co-immunoprecipitation with MT1-MMP antibody or HA-tag antibody, respectively; then HA-Bst-2 or MT1-MMP was checked with related antibodies as described in figures in co-immunoprecipitated protein complexes; (**C**) HT1080 and MDCK cells were seeded and grown on glass coverslips in 6-well plates overnight; then transfected with HA-Bst-2 alone in HT1080 cells, and co-transfected with MT1-MMP and HA-Bst-2 in MDCK cells. After being cultured with GM6001 (5 mM) for 48 h, cells were treated for Immunostaining Confocal Microscopy assay with related antibodies. IP: Immunoprecipitation; IB: Immunoblotting; WB: Western blotting.

To explore the interaction between Bst-2 and MT1-MMP, imunoprecipitation and Western-blot assay were carried out. Cells (HT1080 and MDCK) were cultured in 6-well plates and transfected as in Figure 3A,B. Forty-eight hours later, cells were harvested and lysed for co-immunoprecipitation with either an anti-MT1-MMP antibody or an anti-HA tag antibody. As shown in Figure 3A, endogenous MT1-MMP in HT1080 cells was detected in the protein complex immunoprecipitated by the anti-HA tag antibody; reciprocally, HA-Bst-2 was also detected in the protein complex immunoprecipitated by the anti-MT1-MMP antibody (Figure 3A). In MDCK cells, transient MT1-MMP or HA-Bst-2 were also detected in both protein complexes immunoprecipitated by an anti-HA tag antibody or an anti-MT1-MMP antibody (Figure 3B). These results demonstrated that the interaction between Bst-2 and MT1-MMP happened when co-expressing these two proteins in cells.

To further identify the interaction between Bst-2 and MT1-MMP, Immunostaining and Confocal Microscopy assay was employed to examine the co-localization of Bst-2 and MT1-MMP in cells. HT1080 and MDCK cells were seeded and grown on glass coverslips in 6-well plates overnight and then transfected with HA-Bst-2 alone in HT1080 cells or co-transfected with MT1-MMP and HA-Bst-2 in MDCK cells. After being cultured with GM6001 (5 mM) for 48 h, cells grown on coverslips were treated as described before [28]; the antibodies used here and Immunostaining process were as described in “Materials and Methods”. Confocal microscopy was carried out by a Bio-Rad (Hercules, CA, USA) MRC 1024 system attached to an Olympus microscope (Melville, NY, USA) with a 60X oil objective. The images were processed in Photoshop 7.0 (Adobe, San Jose, CA, USA). From the confocal microscopy results, MT1-MMP mainly localized on cellular membrane in HT1080 cells transfected with pcDNA3.1 and in MDCK cells transfected with only MT1-MMP, and also Bst-2 localized on cellular membrane in MDCK cells transfected with only HA-Bst-2 plasmids; whereas Bst-2 and MT1-MMP predominately co-localized in cytoplasm in granular arrays in HT1080 cells transfected with HA-Bst-2 and in MDCK cells co-transfected MT1-MMP with HA-Bst-2 (Figure 3C). These results demonstrated that MT1-MMP co-localized with Bst-2 and formed granules, and then resulted in both Bst-2 and MT1-MMP mainly transferring into cytoplasm from cellular membrane.

Taken together, MT1-MMP could co-localize and interact with Bst-2 in cells and then form a granular complex trafficking into cytoplasm from membrane, and finally inhibit the membrane tetherin activity of Bst-2.

### 2.4. N-Terminal Domain (NT Domain) of Bst-2 Was Critical in Regulating the Activity of Bst-2 Itself and in Bst-2 Interacting with MT1-MMP

To identify the importance of the N-terminal domain of Bst-2 (NT domain) in regulating the tetherin activity of Bst-2 and in Bst-2 interacting with MT1-MMP, we constructed the NT domain deletion mutant of Bst-2 (HA-Bst-2/ΔNT) to test the role of NT domain in the interaction between Bst-2 and MT1-MMP and in the effect of tetherin activity of Bst-2.

**Figure 4:**
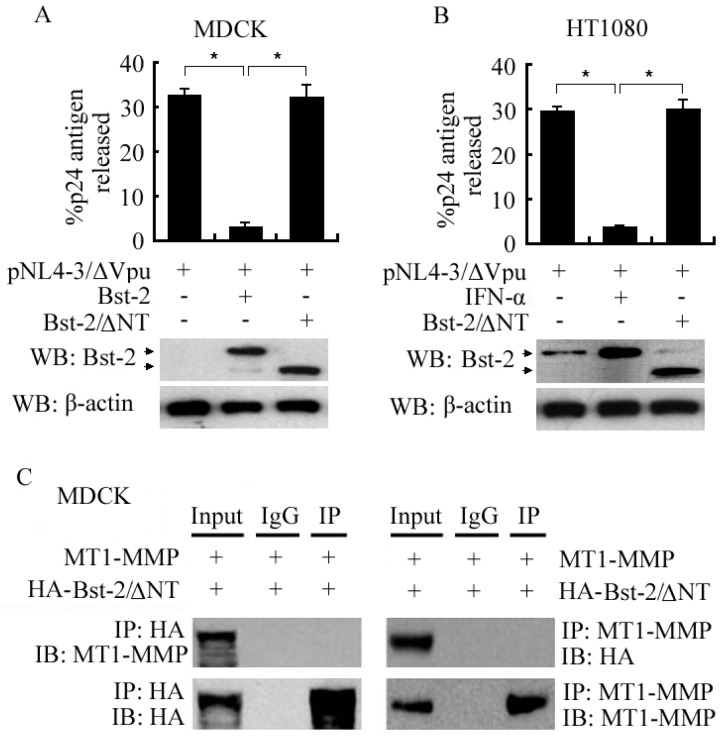
Role of N-terminal domain (NT domain) of Bst-2 in the activity regulation of Bst-2 and the interaction between Bst-2 and MT1-MMP. (**A**,**B**) HT1080 and MDCK cells were divided into two parts and seeded in 6-well plates overnight, and then transfected with plasmids as shown in figures; 48 h later, one part of the culture was used for Bst-2 activity assay by measuring the percentage of released p24 capsids in supernatants in the total concentration of p24 antigen in both supernatants and cell lysates; another part of the cells was lysed for western-blot assay; (**C**) MDCK cells only were seeded in 10-cm plates and transfected with plasmids as shown in figures; 48 h after transfection, cells were harvested and then co-immunoprecipitation assay was employed to check the interaction between HA-Bst-2/ΔNT and MT1-MMP, and further identify the importance of the N-terminal domain of Bst-2 in the interaction of Bst-2 and MT1-MMP. * *p* < 0.01. IP: Immunoprecipitation; IB: Immunoblotting; WB: Western blotting; HA: HA-molecule corresponding to amino acids 98-106.

Cells (HT1080 and MDCK) were seeded in 6-well plates (for virion release and Western-blot assays) and MDCK cells were seeded in 10-cm plates (for co-immunoprecipitation), and cells were then treated with IFN-α and/or transfected/co-transfected with plasmids as indicated in Figure 4. The experimental data of virion release assay and western-blot assay showed that the percentage of released HIV-1 p24 capsid antigen of cells was much decreased by IFN-α-induced Bst-2 (in HT1080 cells) and by transiently expressed Bst-2 (in MDCK cells), but not affected by transiently expressed Bst-2/ΔNT in both HT1080 and MDCK cells (Figure 4A,B). These results demonstrated that the NT-domain of Bst-2 played a critical role in Bst-2 tethering virion on the cellular membrane and blocking virion release from infectious cells.

Co-immunoprecipitation data showed that HA-Bst-2 or MT1-MMP could not be detected in both protein complexes immunoprecipitated by the anti-MT1-MMP antibody or by the anti-HA antibody in MDCK cells when co-transfected with HA-Bst-2/ΔNT and MT1-MMP (Figure 4C). It meant that the interaction between Bst-2/ΔNT and MT1-MMP did not exist; it further indicated that the interaction between MT1-MMP and Bst-2 was totally related to the N-terminal domain of Bst-2.

### 2.5. Importance of the C-Terminal Domain of MT1-MMP in Interacting with Bst-2 and Inhibiting the Tetherin Activity of Bst-2

**Figure 5:**
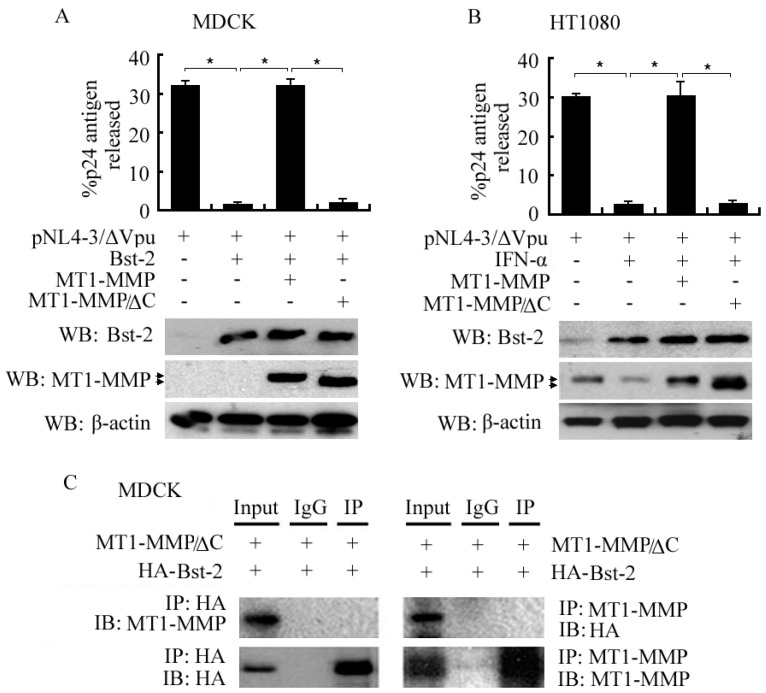
Effect of the C-terminal domain of MT1-MMP on the tetherin activity of Bst-2 and the interaction between these two proteins. (**A**,**B**) HT1080 and MDCK cells were divided into two parts and seeded in 6-plates overnight, and then transfected as indicated in figures; 48 h after transfection, one part of the cultures was used for Bst-2 activity assay as described above; and the other part of the cells was harvested for western-blot assay; (**C**,**D**) MDCK cells were cultured in 10-cm dishes overnight and transfected as in figures; 48 h later, cells were harvested and then co-immunoprecipitation assay was employed to check the interaction between HA-Bst-2 and MT1-MMP/ΔC, and further identify the importance of the C-terminal domain of MT1-MMP in the interaction of Bst-2 and MT1-MMP. * *p* < 0.01.

To document the role of the C-terminal domain of MT1-MMP in affecting the activity of Bst-2, cells (HT1080 cells and MDCK cells) were seeded in 6-well plates (for virion release assay and western-blot assay) and MDCK cells seeded in 10-cm dishes (for co-immunoprecipitation). Twenty-four hours later, cells were treated with IFN-α and/or transfected/co-transfected with plasmids as indicated in Figure 5. From our data, the fractional p24 antigen release of cells with pNL4-3/ΔVpu was much higher than that of cells with pNL4-3/ΔVpu plus treatment of IFN-α in HT1080 cells than that of cells with pNL4-3/ΔVpu plus transfected Bst-2 in MDCK cells; then, the inhibition of p24 antigen release by Bst-2 (including IFN-α-induced Bst-2 and transiently expressed Bst-2) was reversed by co-transfected MT1-MMP, but not by co-transfected MT1-MMP/ΔC ( the deletion mutant of the C-terminal domain of MT1-MMP) in both HT1080 cells and MDCK cells (Figure 5A,B). It indicated that C-terminal domain of MT1-MMP was important for MT1-MMP in inhibiting the tetherin activity of Bst-2 and reversing the inhibition of virion release by Bst-2 from infectious cells.

Furthermore, co-immunoprecipitation data showed that HA-Bst-2 or MT1-MMP could not be detected in both protein complexes immunoprecipitated by anti-MT1-MMP antibody or by anti-HA antibody in MDCK cells after co-transfection with HA-Bst-2 and MT1-MMP/ΔC (Figure 5C). It implicated that the interaction between MT1-MMP/ΔC and Bst-2 could not happened; and the interaction between MT1-MMP and Bst-2 was totally related to the C-terminal domain of MT1-MMP.

In short, the N-terminal domain of Bst-2 and the C-terminal domain of MT1-MMP were pivotal in the regulation of the tetherin activity of Bst-2 and the interaction between these two proteins.

### 2.6. Role of the N-Terminal Domain of Bst-2 in Down-Regulating the Activity of MT1-MMP

It is well known that MT1-MMP plays a positively important role for cell growth in type I collagen gels and for cancer cell migration via activating proMMP2 into active MMP2 [9,10]. Furthermore, our previous report showed that Bst-2 inhibited the activities of MT1-MMP and decreased the activation of proMMP2 in HT1080 and MDCK cells [29]. Our data here precisely demonstrate that the protein-protein interaction happened only between MT1-MMP and Bst-2, and not between MT1-MMP and Bst-2/ΔNT (Figure 4C). Accordingly, mutant Bst-2/ΔNT should not subsequently inhibit the activity of MT1-MMP and block the enhancement of MT1-MMP on cell growth and cell migration. To identify this point, an activation assay of proMMP2, cell growth in 3-D type I collagen gels and cell migration experiments were employed.

**Figure 6:**
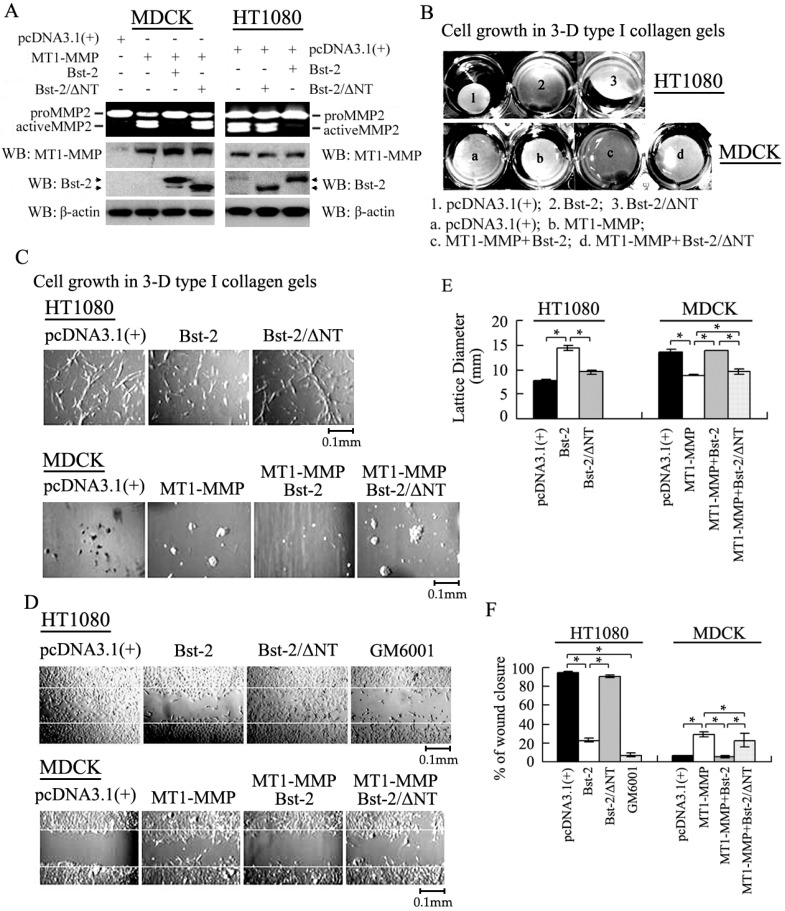
Effect of the N-terminal domain of Bst-2 on MT1-MMP activity and MT1-MMP-promoted cell growth and migration. MDCK cells and HT1080 cells were seeded in 6-well plates overnight and transfected as described in figures; (**A**) 24 h after transfection, medium were changed into DMEM with 5% FBS; another 24 h later, culture medium were collected and centrifuged at 12,000 rpm for 10 min, and then subjected to zymography gel assay; then, cells were harvested and lysed for Western-blot assay; (**B**,**C**) also 24 h after transfection, the culture containing the same amount of cells (1 × 10^3^) was mixed with 500 mL of type I collagen (2.5 mg/mL) and allowed to gel at 37 °C in 24-well plates to give rise to three-dimensional collagen lattice; it was then cultured with new medium for one week. The gel lattices containing cells and cells grown in gel lattice were photographed with a video camera; (**D**) after transfected HT1080 and MDCK cells growing to almost confluency in 6-well plates; a scratch wound assay was employed to check the effect of wide type Bst-2 and Bst-2/ΔNT on cellular migration ability induced by MT1-MMP; (**E**,**F**) the quantification of the 3-D gel lattices (lattice diameter) in (**B**) and the percentages of wound closure in wound healing assays (the quantification of wound healing assays) in (**D**). Error bars indicate s.d. among three individual experiments. * *p* < 0.05.

For activation assay of proMMP2, cells (HT1080 cells and MDCK cells) were seeded in 6-well plates overnight and transfected as described in Figure 6A. Forty-eight hours after transfection, culture medium was collected and centrifuged for zymography gel assay; then, cells were harvested and lysed for western-blot assay. From the experimental data, the amount of active MMP2 (activated from proMMP2 by MT1-MMP) was great decreased by transiently over-expressed Bst-2, rather than by transiently over-expressed Bst-2/ΔNT in both HT1080 cells and MDCK cells. Protein levels of MT1-MMP, from western-blot data, were not changed in these two cell lines (Figure 6A). These results demonstrated that the N-terminal domain of Bst-2 was critical for MT1-MMP to activate proMMP2 into active MMP2; they also indicated that the interaction that happened between Bst-2 and MT1-MMP was dependent on the N-terminal domain of Bst-2.

As we know, type I collagen, a component of extracellular matrix (ECM), was the substrate of MT1-MMP and MMP2 (activated by MT1-MMP) expressed in HT1080 cells [10,11]. From our data of HT1080 cells growing in 3-D type I collagen gels, we found that the 3-D gel lattices containing cells transfected with Bst-2 were bigger than the lattices containing cells transfected with pcDNA3.1 and Bst-2/ΔNT (Figure 6B, upper panel). It indicated that type I collagen was degraded by MT1-MMP and MMP2 (expressed by HT1080 cells) in cells transfected with pcDNA3.1 and Bst-2/ΔNT rather than in cells transfected with Bst-2. It meant that the activities of MT1-MMP and MMP2 were inhibited by transfected Bst-2 rather than by Bst-2/ΔNT. Further, growth of HT1080 cells in 3-D type I collagen gels was inhibited by transfected Bst-2 when compared to the cells transfected with pcDNA3.1 and Bst-2/ΔNT (Figure 6C, upper panel). In MDCK cells, the 3-D gel lattices containing cells transfected with MT1-MMP and with co-transfected MT1-MMP and HA-Bst-2/ΔNT were also obviously smaller than those lattices containing cells transfected with pcDNA3.1 and with co-transfected MT1-MMP and HA-Bst-2 (Figure 6B, down panel). Cell growth in 3-D type I collagen gels was enhanced by MT1-MMP and this enhancement was also greatly inhibited by Bst-2 rather than by Bst-2/ΔNT (Figure 6C, down panel). These results indicated that the N-terminal domain of Bst-2 was a key fragment in Bst-2 inhibiting the activity of MT1-MMP and blocking cell growth in type I collagen gels.

Additionally, the effect of Bst-2 and Bst-2/ΔNT on the role of MT1-MMP in cell migration was also examined. In the scratch-wound test, the migration of HT1080 cells was greatly inhibited by transfected Bst-2 (the same as the positive control of the GM6001 treatment sample), while not affected by transfected Bst-2/ΔNT, when compared with cells transfected with pcDNA3.1 (Figure 6D, upper panel). In the MDCK cell line, the migration of cells transfected with MT1-MMP and co-transfected MT1-MMP with HA-Bst-2/ΔNT was greater than that of cells transfected with pcDNA3.1 and co-transfected MT1-MMP with HA-Bst-2 (Figure 6D, down panel). These results demonstrated that the enhancement of cell migration by endogenous or transient MT1-MMP (in HT1080 or MDCK cells) was inhibited by Bst-2, rather than by Bst-2/ΔNT.

All these results indicated that membrane protein Bst-2 inhibiting MT1-MMP activity on promoting cell growth and cell migration was also via the interaction between these two proteins; and the role of the N-terminal domain of Bst-2 was important in their interaction and in Bst-2 inhibiting the function of MT1-MMP/proMMP2/MMP2 pathway axis.

### 2.7. Discussion

Bst-2 and MT1-MMP are both membrane proteins. The cytoplasmic tail of Bst-2 is the N-terminal domain. Bst-2 mainly functions as a tetherin to restrict virus releasing from infected cells onto cellular membrane [3]. The cytoplasmic tail of MT1-MMP is the C-terminal domain. MT1-MMP/proMMP2/MMP2 axis is very important in cell growth and metastasis [9,10]. We previously reported that Bst-2 could inhibit the activity of MT1-MMP through their interaction and then block the function of the MT1-MMP/proMMP2/activeMMP2 pathway in MDCK and HT1080 cell lines [29]. In this study, we use the MDCK cells (they have no endogenous MT1-MMP expression) and HT1080 cells (they have a little endogenous MT1-MMP expression and higher expression of Bst-2 is induced by IFN-α in these cells) to explore the molecular mechanism of MT1-MMP inhibiting the activity and bio-function of Bst-2. From the results, we find that MT1-MMP reversely inhibits the activity and bio-function of Bst-2 through their interaction. The cytoplasmic tails of Bst-2 (N-terminal domain) and MT1-MMP (C-terminal domain) play critical roles in both the interaction of MT1-MMP and Bst-2 and the inhibition of the activity/bio-function of Bst-2 by MT1-MMP. Furthermore, we find that the N-terminal domain of Bst-2 is also important for Bst-2 to block the activity and bio-function of the MT1-MMP/proMMP2/activeMMP2 pathway.

Bst-2 and MT1-MMP are constitutively expressed transmembrane proteins in many types of cell lines. It has been reported that Bst-2 and MT1-MMP can be endocytosed and circulated, respectively, between membrane and cytoplasm mediated by their cytoplasmic tails and other proteins [7,22]. The endocytosis and circulation of Bst-2 and MT1-MP are important for these two proteins to carry out their activities and bio-functions [4,30]. The data of immunostaining and confocal microscopy assay here show that MT1-MMP interacts with Bst-2 and then forms granules (complex vesicles) mainly locating in cytoplasm. It demonstrates that the co-endocytosis of Bst-2 and MT1-MMP happens here and then decreases the amounts of Bst-2 and MT1-MMP on the cellular membrane, and finally results in the activities of both Bst-2 and MT1-MMP on the cellular membrane being down-regulated. This conclusion indicates that we have characterized a new way of activity regulation of both Bst-2 and MT1-MMP through their co-endocytosis pathway. Clathrin was reported to relate to the endocytosis of both Bst-2 and MT1-MMP, respectively [7,31]. Together with our results, clathrin may be contained in the granule complex formed by Bst-2 and MT1-MMP, and may play a role in the interaction between Bst-2 and MT1-MMP.

Bst-2 protein needs to polymerize to form dimers on membrane for executing its bio-functions; the N-terminal domain of Bst-2 is a key domain mainly related to the polymerization of Bst-2 protein [30,32]. From our experimental data, we find that the N-terminal domain of Bst-2 is important in the interaction between Bst-2 and MT1-MMP, co-endocytosis of Bst-2 and MT1-MMP and activity regulation of Bst-2 by MT1-MMP (Figure 3, Figure 4 and Figure 5). Based on this, MT1-MMP does not only carry out the co-endocytosis with Bst-2 and activity inhibition of Bst-2 via their interaction, but may prevent the polymerization of Bst-2 on membrane. It indicates that the N-terminal domain of Bst-2 may be important in MT1-MMP blocking the dimerization of Bst-2 protein on cellular membrane.

In this study, the experimental data show that wild-type Bst-2 interacts with MT1-MMP and forms complex trafficking into cytoplasm from cellular membrane, whereas the interaction dose not happened to N-terminal deletion mutant Bst-2/ΔNT. The wild-type Bst-2 (including IFN-α-induced Bst-2 and transiently expressed Bst-2) inhibits the cell growth in 3-D type I collagen gels and cell migration *in vitro* by inhibiting the activity of MT1-MMP, but the mutant Bst-2/ΔNT has no effect on MT1-MMP (Figure 6). These results elucidate that the N-terminal domain of Bst-2 is important in the interaction between Bst-2 and MT1-MMP and plays a key role in the activity and bio-function regulation of MT1-MMP and Bst-2 itself (Figure 4 and Figure 6). As we know, the N-terminal domain of Bst-2 structurally contains three key sequences, including the YXY sequence, the KXXK sequence and the D/GDIWK sequence. The three sequences are all critical for the function of this domain [5]. Because of this, the specific and detailed importance of the three key sequences in the interaction of Bst-2 and MT1-MMP needs to be further investigated in the next study.

It is reported that the cytoplasmic tails of MT1-MMP (C-terminal domain) are not only important for MT1-MMP to carry out its activity and bio-function, but for MT1-MMP to interact directly or indirectly with other proteins [21,22]. In this study, we find that the deletion mutant of the cytoplasmic tail of MT1-MMP (MT1-MMP/ΔC) cannot interact with wild-type Bst-2; and this interaction removal results in disappearing the inhibition of the activity and bio-function of Bst-2 by MT1-MMP (Figure 5). It illustrates that the C-terminal domain of MT1-MMP is also critical for MT1-MMP to interact with Bst-2 and to regulate the activity of Bst-2. Altogether, our results indicate that both the C-terminal domain of MT1-MMP and the N-terminal domain of Bst-2 are crucial not only in mediating the interaction between Bst-2 and MT1-MMP, but in regulating the activities and bio-functions of these two proteins themselves.

Summarily, our studies demonstrate that the interaction between two membrane proteins, Bst-2 and MT1-MMP, actually happens and the cytoplasmic tails, both the N-terminal domain of Bst-2 and the C-terminal domain of MT1-MMP, play crucial roles in the interaction. The interaction between Bst-2 and MT1-MMP is important in MT1-MMP regulating the tetherin activity of Bst-2 and also in Bst-2 regulating the activity of the MT1-MMP/proMMP2/MMP2 pathway. These findings may give us some clues in the clinic to explore new methods and drugs in prohibiting cancer cell migration and in preventing some transmission of viral infections, like HIV infections.

## 3. Materials and Methods

### 3.1. Cell Culture and Transfection

Tissue culture medium and related reagents were purchased from BRL-GIBCO Co. Ltd., Grand Island, NY, USA. Both of the cell lines, MDCK and HT1080, were obtained from American Type Culture Collection (ATCC, Manassas, VA, USA) and subcloned subsequently. Subline MDCK-µmn [30] is epithelial-like in cell shape and grows well in DMEM medium and was used throughout the experiments. Complete DMEM (dulbecco s minimum essential medium) medium contains 10% FBS (fatal bovine serum), 2 mM l-glutamine and 50 units/mL streptomycin/penicillin. HT1080 cells were maintained as described [33,34]. All cells were cultured in a growth chamber with 5% CO_2_/95% air at 37 °C.

For transfection, cells were seeded and cultured overnight, and then the wild-type and mutant DNA constructs were transfected into cells by Lipofectamine 2000 using protocols as described by the manufacturer (Invitrogen Inc., Carlsbad, CA, USA).

### 3.2. Plasmids, Antibodies and Chemicals

A pcDNA3.1(+)/MT1-MMP and pcDNA3.1 (+)/MT1-MMP/ΔC (C-terminal domain deletion mutant) were constructed as described previously [14,15] and kept in our lab. A pcDNA3.1 (+)/HA-Bst-2 was constructed by ourselves described as previously [29]. A pcNDA3.1 (+)/HA-Bst-2/ΔNT (deleted 17aa of N-terminal of Bst-2 protein) was constructed by using one-step mutagenesis kit. The PCR products were connected into a pcDNA3.1 (+) uni-HA vector which was constructed and kept by our lab (HA tag located just behind Xho I site). The mutant pNL4-3/ΔVpu of provirus plasmid pNL4-3, which was originally obtained from the National Institute of Health (NIH) AIDS Research & Reference Reagent Program, was generated by PCR mutagenesis and contained a deletion of the first 10 nucleotides of the Vpu open-reading frame and saved by our lab (this mutant plasmid was Vpu-defective).

A mouse monoclonal MT1-MMP antibody, a rabbit polyclonal HA-tag antibody and a rabbit polyclonal Bst-2 antibody were purchased from Santa Cruz Biotechnology (Carlsbad, CA, USA). Anti-β-actin antibody was bought from Cell Signaling Technology (Danvers, MA, USA). Type I collagen was from Collaborative Research (Bedford, MA, USA). A co-immunoprecipitation kit was bought from Promega Inc. (Madison, WI, USA). Alexa Fluor^®^ 488 goat anti-mouse IgG and Alexa Fluor^®^ 594 goat anti-rabbit IgG were got from Invitrogen Inc. IFN-α (type I interferon alpha) was purchased from Sigma-Aldrich Inc. (Saint Louis, MO, USA) and used at a final concentration 2000 U/mL in all experiments. Other chemicals were purchased from Sigma-Aldrich or Fisher (Pittsburgh, PA, USA).

### 3.3. Zymography, Western Blotting and Immunoprecipitation

We carried out zymography gel assay as described before [35]. Briefly, cells were seeded and cultured in a 12-well plate and transfected as indicated in figures. Twenty-four hours after transfection, medium was changed into DMEM containing 5% FBS. After culturing another 24 h, the medium were collected and centrifuged at 12,000 rpm for 10 min, and then subjected to SDS-PAGE gel impregnated with 1 mg/mL gelatin as described previously [36]. The gels were incubated at 37 °C overnight, stained with Coomassie Blue, destained, and then scanned.

For western-blot and co-immunoprecipitation experiments, transfected cells were cultured in medium with 5 µM of MMPs inhibitor GM6001 to avoid their auto-degradation. At 48 h after transfection, cells were harvested and centrifuged at 4 °C. For the western-blot experiment, cell pellets were lysed in lysis buffer (50 mM Tris-HCl, pH 7.5, 150 mM NaCl, 0.25% sodium deoxycholate, 0.1% Nonidet P-40 and a mixture of protease inhibitors) and cleared by centrifugation. The supernatants were used for Western-blot assay with the antibodies indicated in the figures. For the co-immunoprecipitation experiment, the harvested cell pellets were lysed and treated by using a Co-immunoprecipitation Kit from Promega Inc. as described in its introductions. Western-blot analysis of co-immunoprecipitation was done with specific antibodies of proteins as described in the figures.

### 3.4. Reverse Transcription PCR (RT-PCR) and Quantitative Real-Time PCR (qPCR)

Total RNA of cells was extracted by using the Trizol reagent (Life Technologies, Inc., Burlington, ON, Canada) handled as per the manufacturer’s instructions. 2 μg of total RNA were mixed with a reaction mixture (30 mM KCl, 8 mM MgCl_2_, 10 mM DTT, 100 ng oligo-dT 12–18, 40 units of RNase inhibitor, 50 mM Tris-HCl, pH 8.3 and 1 mM deoxynucleotide triphosphates) and eight units of avian myeloblastosis virus reverse transcriptase (Pharmacia Biotech, Baie D’Urfe, QC, Canada). The reverse transcription was finished by incubating the mixture for 10 min at 25 °C, then for 45 min at 42 °C, and finally for 5 min at 95 °C. The PCR primers used were as follows: for Bst-2: sense 5′-CCTGCTCGGCTTTTCGCTTGAACAT-3′, antisense 5′-CGGAGGGAGGCTCTGGAGGGAGAC-3′; for β-Actin: sense 5′-GGACTTCGAGCAAGAGATGG-3′, antisense 5′-AGCACTGTGTTGGCGTACAG-3′. The cDNA was amplified using the PCR method and amplified products were analyzed by electrophoresis in 1.0% agarose gels.

For the qPCR experiment, total RNA was extracted from cells using Trizol reagent (Invitrogen, Carlsbad, CA, USA) and reverse-transcribed to cDNA by using M-MLV Reverse Transcriptase (Promega) with oligo(dT) 12–18 primers. The cDNAs were quantified and qRT-PCR was carried out by using the ABI PRISM 7500 Real-Time PCR System (Applied Biosystems, Foster City, CA, USA). Each well of the 96-well plate contained 20 μL reaction mixture with 10 μL Power SYBR Green PCR master mix (Applied Biosystems), 1 μL template and1 μL of each primer (5 μmol/L). Primer sequences for cDNAs were as follows: BST-2 (sense: 5′-CTGCAACCACACTGTGATG-3′, antisense: 5′-ACGCGTCCTGAAGCTTATG-3′), GAPDH (sense: 5′-GTCCACTGGCGTCTTCACCA-3′, antisense: 5′-GTGGCAGTGATGGCATGGAC-3′). The GAPDH quantification was used to normalize the starting amount of total RNA for quantifying BST-2 mRNA.

### 3.5. Growth of MDCK and HT1080 Cells in Three-Dimensional Type I Collagen Lattice

Cells (MDCK and HT1080) were seeded in 6-well plates and transfected with plasmids as indicated in figures using Lipofectamine 2000. Twenty-four hours after transfection, the cultures containing the same amount of cells (1 × 10^3^) were mixed with 500 μL of type I collagen (2.5 mg/mL), then incubated in 24-well plates at 37 °C to form 3-D (three-dimensional) collagen lattice. Fresh complete medium was added into the wells and changed every 2 days. After one week, the gel lattice containing cells and cells grown in collagen gels were photographed by a video camera as described previously [36].

### 3.6. Migration Assay of MDCK and HT1080 Cells

Cell motility was tested using a scratch-wound assay [30]. Equal numbers of MDCK or HT1080 cells were cultured in 6-well plates with DMEM and transfected with plasmids as indicated in figures. When cells grew to almost confluency in the wells, a scratch was made along the axis of each well using a pipette tip. Cells were washed three times with PBS buffer, and then cultured with new DMEM medium. Sixty hours later, migration of cells into the scratch was photographed by a video camera as described previously [36].

### 3.7. Immunostaining and Confocal Microscopy Assay

HT1080 and MDCK cells were grown on glass coverslips overnight and then transfected with the plasmids as indicated in figures. After being cultured for 48 h in the full medium with GM6001 (5 μM), cells were fixed with 4% polyformaldehyde for 20 min and then incubated with PBS containing 0.1% Triton X-100 for 5 min. After blocking with 3% goat serum in PBS, 0.2 μg/mL anti-MT1-MMP antibody (mouse) and anti-HA tag antibody (rabbit) were added to the cells, respectively; then cells were incubated at 4 °C overnight. Secondary antibodies, including Alexa Fluor^®^ 594-labeled goat anti-rabbit IgG and Alexa Fluor^®^ 488-labeled goat anti-mouse IgG, were used to bind and detect the primary antibody. Confocal microscopy assay was carried out in the Biomedical Image Processing laboratories using a Bio-Rad MRC 1024 system attached to an Olympus microscope (Melville, NY, USA) with a 60× oil objective. The images were processed in Photoshop 7.0 (Adobe, San Jose, CA, USA).

### 3.8. Virion-Release Assay

The same amount of cells, HT1080 and MDCK, were seeded in 6-well plates overnight, and then transfected with pNL4-3/ΔVpu or co-transfected with pNL4-3/ΔVpu and the plasmids as indicated in figures. Forty-eight hours later, a HIV-1 p24 antigen capture ELISA kit (Perkin-Elmer, Waltham, MA, USA) was used to check the concentration of viral capsid protein in the supernatants of culture medium that were first clarified by centrifugation at 400× *g* and the concentration of viral capsid protein in detergent lysates (0.5% Triton-X-100 in PBS) of the cultured cells. The fractional release of p24 capsid was calculated as the percentages of p24 antigen concentration of the supernatants in the total p24 antigen concentration of both the supernatants and the cell lysates.

### 3.9. Statistical Analysis

Data are expressed as mean ± SD (standard deviation) from at least three experiments, and statistical analyses were performed by Student’s *t* test using the Microsoft Excel computer program. *p* < 0.05 was considered as statistically significant.

## 4. Conclusions

This study shows a novel mode of regulation of membrane protein Bst-2 by membrane protein MT1-MMP. There are three new findings regarding this regulation mode: (1) MT1-MMP down-regulates the activity and bio-function of Bst-2 via their interaction and co-endocytosis; (2) both cytoplasmic domains of Bst-2 (N-terminal domain) and MT1-MMP (C-terminal domain) play important roles in their interaction; and (3) the N-terminal domain of Bst-2 is also important in Bst-2 inhibiting the activity of MT1-MMP and bio-function of the MT1-MMP/proMMP2/MMP2 pathway.

## Supplementary Materials

Supplementary materials can be found at http://www.mdpi.com/1422-0067/17/6/818/s1.
