# Supplementary material for: MT1-MMP Inhibits the Activity of Bst-2 via Their Cytoplasmic Domains Dependent Interaction
Source: Int J Mol Sci. 2016 May 26;17(6):818. doi: 10.3390/ijms17060818 (PMC4926352; doi:10.3390/ijms17060818)
Supplement: Supplementary file 1 [file ijms-17-00818-s001.pdf]

Supplementary Materials: MT1-MMP Inhibits the Activity of Bst-2 via Their Cytoplasmic Domains Dependent Interaction

Long Fan, Li Liu, Cuicui Zhu, Qingyi Zhu, Shan Lu and Ping Liu

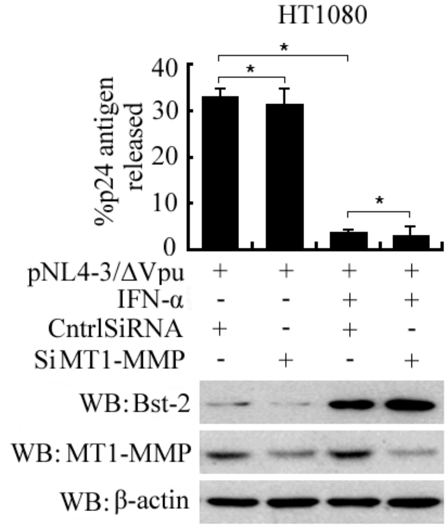

Figure S1. HT1080 cells were transfected as indicated in figure. Forty-eight hours later, culture supernatants and cells were harvested for virus release assay and western-blot assay. * $p < 0.01$.
